# Autologous bone marrow cell transplantation in the treatment of HIV patients with compensated cirrhosis

**DOI:** 10.1042/BSR20191316

**Published:** 2020-06-23

**Authors:** Baochi Liu, Mingrong Cheng, Xiaodong Chen, Lei Li, Yanhui Si, Shijia Wang, Ying Wang, Yufang Shi

**Affiliations:** 1 Department of Surgery, Shanghai Public Health Clinical Center, Fudan University, Shanghai 201508, China; 2 Department of General Surgery, Jiangqiao Hospital of Jiading District, Jiading Branch of Shanghai First People’s Hospital, Shanghai 201803, China; 3 Key Laboratory of Stem Cell Biology, Institute of Health Sciences, Shanghai Institutes for Biological Sciences, Chinese Academy of Sciences, Shanghai 200031, China

**Keywords:** Autologous bone marrow cell transplantation, decompensated liver cirrhosis, human immunodeficiency virus, stem cell

## Abstract

Liver stem cell therapy is a promising tool to improve decompensated liver cirrhosis (DLC). Especially in patients infected with human immunodeficiency virus (HIV), the condition of the liver may be aggravated by antiretroviral therapy. A total of 21 patients diagnosed with DLC and HIV infection were divided into two groups as follows: those who received (combination therapy group, 14 patients) and those who did not receive (routine therapy group, 7 patients) bone marrow cell transplantation through the portal vein. Two patients died of surgery-related complications in the combination therapy group. The results showed that the survival rate was 85.7% in the combination therapy group after 2 years of follow-up, which was significantly higher than the 14.3% in the conventional therapy group (*P*<0.01). After treatment, the liver function score decreased significantly in the combination therapy group at 1 (*t* = 4.276, *P* = 0.000), 3 (*t* = 9.153, *P* = 0.000), and 12 (*t* = 13.536, *P* = 0.000) months, the levels of albumin were significantly increased, and the total bilirubin level and prothrombin time were significantly reduced or shortened as compared with the routine therapy group (*P*<0.05 or <0.01). The white blood cell count, hemoglobin, platelet count, and CD4+ and CD8+ levels were significantly higher in the combination therapy group at different time points as compared with the routine therapy group (*P*<0.05 or <0.01). In summary, the combination therapy is effective in HIV-infected patients with DLC and useful for the recovery of liver function and cellular immune function but may increase the risk of severe complications after surgery.

## Introduction

Decompensated liver cirrhosis (DLC) is a stage in the progression of liver cirrhosis. It is associated with functional impairment of liver and portal hypertension. The main causes of liver cirrhosis include hepatitis B virus (HBV) [1,2], alcohol [3], hepatitis C virus (HCV) [4], and autoimmune liver disease. Stem cell therapy has generated great interest due to an increased incidence of chronic DLC and the shortage of organ donors for liver transplantation [5]. Moreover, DLC has a poor prognosis with elevated short-term mortality, even in steroid-treated patients. Thus, early liver transplantation can be an option for selected patients but is unavailable to the majority of patients [6]. Stem cell-based strategies have been tested as an alternative to organ transplantation.

Although anti-fibrotic drugs have been developed, their efficacy on liver disease is limited. The studies on bone marrow cells (BMCs) have facilitated the application of cellular therapeutics. Furthermore, hepatocytes from bone marrow (BM) have been found in mouse and human livers after BMC transplantation, suggesting that BMCs contribute to liver regeneration [7–10]. Also, the effect of BMCs injected into fibrotic or injured livers has been investigated. However, BMCs must undergo *in vitro* selection and culture for clinical application. Therefore, a clinical trial using mesenchymal stem cells (MSCs) cannot be started until safety during *in vitro* manipulation is ensured [11]. Although early studies suggested the transdifferentiation of BMCs or MSCs into hepatocytes, the underlying mechanism remains poorly understood. The condition of the liver may be aggravated by antiretroviral therapy (ART), especially for patients infected with human immunodeficiency virus (HIV), thereby necessitating a feasible treatment. The present study enrolled 21 patients who were infected with HIV and developed DLC from April 2010 to June 2016. All patients underwent antiretroviral and liver treatment. Of the 14 patients, 12 underwent splenectomy combined with BMC transplantation through the portal vein. The BMC infusion promoted the reestablishment of the liver and immune system.

## Materials and methods

### Patient information

A total of 17 male and 4 female patients, aged 26–56 (average: 40.3) years, were recruited in the present study. All patients were diagnosed with DLC and HIV and underwent treatment at the Shanghai Public Health Clinical Center, China. Of these, 16 patients developed liver cirrhosis due to HBV infection and 5 due to HCV infection. The present study was approved by the Ethics Committee of the Shanghai Public Health Clinical Center, and all subjects provided informed consent before participation in the present study.

### Clinical findings

Decompensated cirrhosis was identified based on the presence of one of the following clinical characteristics: ascites, bleeding varices, encephalopathy, use of spironolactone without alternative indication, or explicit mention of decompensated cirrhosis. The patients were assessed for serum biochemical indexes, including total serum bilirubin (12.9–56.9 µmol/l), white blood cells (WBCs; 2.1–3.35 × 10^9^/l), hemachrome (56.9–125 g/l), thrombocyte (16–10^6^ × 10^9^/l), alanine aminotransferase (26–47 U/l), aspartate aminotransferase (17–65 U/l), CD4+ T lymphocytes (61–303 cells/µl), CD8+ T lymphocytes (174–324 cells/µl), and CD4+/CD8+ (0.27–1.71). Moreover, 17 patients were graded as Child–Pugh–Turcotte class B (a score of 7–9 on a scale of 5–15, with higher values indicating advanced liver disease) and 4 as class C (score ≥10). Among all patients, 16 presented a history of upper gastrointestinal tract hemorrhage.

### Therapeutic intervention

All patients underwent routine therapy, including diuresis, liver protection, yellowing, albumin supplementation, prevention of gastrointestinal bleeding, ART regimen (lamivudin 300 mg/day, tenofovir 300 mg/day, and lopinavir 400 mg/day), and liver treatment (sofosbuvir 300 mg/day, for HCV infection). Furthermore, 12 patients from the cohort consisting of 14 underwent splenectomy and autologous BMC transplantation through the portal vein and were classified as the combination therapy group. Seven patients who refused splenectomy and received only routine therapy were classified as the routine therapy group because this treatment was carried out only at Shanghai Public Health Clinical Center (Shanghai, China); thus, its efficacy needs to be evaluated.

### Splenectomy and autologous BMC transplantation

General anesthesia was administered to all patients. Nodular cirrhosis and enlarged spleen were observed. The patients exhibited 500–3500 ml of ascites. Venous access ports were inserted through the right omental vein and subcutaneously implanted in the abdomen. The spleen and a piece of liver were resected for pathological examination. One week after the surgery, 20 ml BMC was obtained by a puncture at the anterior superior iliac spine, which was then injected into the vein via venous access ports. Eventually, the venous access ports were filled with 5 ml of sterile heparinized saline to prevent the formation of clots. The same protocol was followed for autologous BMC infusion at 1 month and 3 months after the surgery.

### Blood biochemical analysis

Before treatment and 1, 3, 12, and 24 months after the treatment, the serum samples obtained from the patients were analyzed using the DA 3500 Discrete Automatic Chemistry Analyzer (Fuji Medical System Co. Ltd, Tokyo, Japan) to evaluate the serum biochemical indexes, including serum prothrombin time, albumin, and total bilirubin. A Sysmex XS-800i Automatic Blood Cell Analyzer (Sysmex Shanghai Ltd, Shanghai, China) was used to evaluate the routine blood tests such as WBC count, hemoglobin, and platelets.

### Flow cytometry analysis

Five ml blood sample was collected in ethylenediaminetetraacetic acid (EDTA)-coated tubes. Red blood cells were lysed by adding 5 ml of ammonium chloride-potassium lysis buffer (0.16 M NH_4_Cl, 10 mM KHCO_3_, 0.13 mM EDTA; pH 7.2) for 5 min on ice, followed by washing two times with phosphate-buffered saline. Single-cell suspensions (1  ×  10^6^ cells/sample) were incubated with appropriate antibodies for 45  min at 4°C. Then, the cells were washed and detected using a FACS Canto II flow cytometer (BD Biosciences, CA, U.S.A.). Data were analyzed using FlowJo software (Tree Star, OR, U.S.A.). The following antibodies were purchased from BioLegend (CA, U.S.A.): fluorescein isothiocyanate-labeled anti-CD3, PE-labeled anti-CD4, and APC-labeled anti-CD8.

### Statistical analysis

All patients were followed up for more than 2 years. Descriptive data are expressed as mean ± standard deviation (mean±SD), whereas enumeration data are expressed as a rate. The differences between the samples were analyzed using the rank-sum analysis. Survival analysis was used to compare the survival rates between the two groups. *P*-value <0.05 was considered statistically significant.

## Results

### Comparison of pre-treatment baseline data between the two groups

The baseline data of the two groups are shown in Table 1. No statistical differences were detected in the age, weight, body mass index, CD4+, CD8+, prothrombin time, albumin, total bilirubin, WBCs, platelet, hemoglobin, and liver function between two groups (*P*>0.05).

**Table 1:** Comparison of baseline parameters before the treatment in both groups (mean ± SD)

Groups	Age (years)	Weight (kg)	Body mass index (kg/m^2^)	CD4+ (cells/µl)	CD8+ (cells/µl)	Prothrombin time (s)	Albumin (g/l)	Total bilirubin (g/l)	White blood cells (10^9^/l	Platelet (10^9^/l)	Hemoglobin (g/l)	Liver function (score)
Combination therapy	40.33 ± 10.44	62.35 ± 13.62	25.67 ± 9.64	239.92 ± 137.19	314.83 ± 168.03	18.55 ± 1.41	33.28 ± 3.78	25.92 ± 17.87	2.53 ± 1.17	72.58 ± 65.12	87.92 ± 24.46	8.00 ± 1.13
Routine therapy	45.43 ± 7.65	64.87 ± 17.68	27.93 ± 11.67	184.71 ± 104.04	265.57 ± 131.49	18.14 ± 0.86	32.09 ± 2.72	17.33 ± 6.25	2.67 ± 1.50	89.43 ± 79.52	91.57 ± 25.00	7.29 ± 0.49
*t*	1.122	0.362	0.473	0.918	0.663	0.687	0.731	1.514	0.224	0.502	0.310	1.577
*P*	0.278	0.721	0.642	0.372	0.516	0.501	0.475	0.151	0.825	0.622	0.762	0.133

### Efficacy of combination BMC transplantation with splenectomy and follow-up

**Figure 1:**
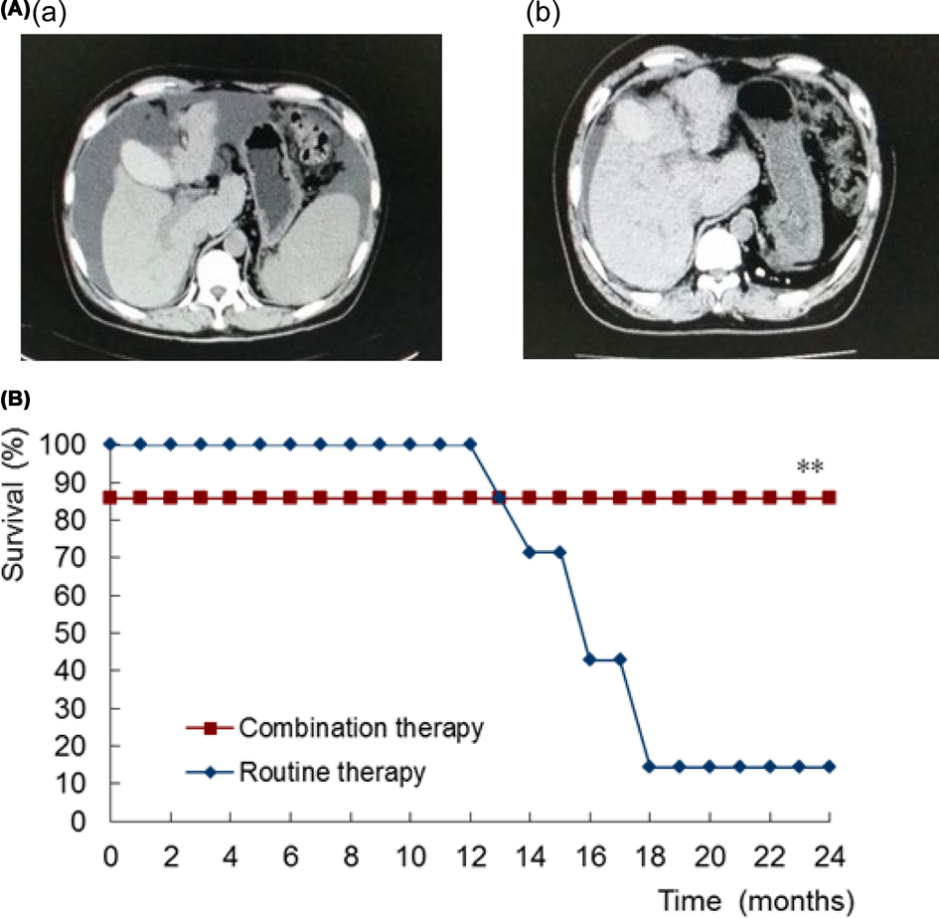
Computed tomography and survival analysis after 2 years of follow-up (**A**) Computed tomography before and after treatment. (**a**) The liver was obviously atrophic with abundant ascites in the abdominal cavity and enlarged spleen. (**b**) The liver size increased significantly and the ascites almost disappeared after 2 years of follow-up. (**B**) Comparison of survival analysis after 2 years of follow-up. Compared with the routine therapy group, **P<0.01.

In the combination therapy group consisting of 14 patients, 2 showed intraperitoneal hemorrhage on the day of surgery. The surgical wounds in these patients oozed profusely when the operation was repeated. The bleeding wounds were treated with suture ligation and argon laser knife. Both patients died of abdominal bleeding and liver failure 2 days after the surgery. All other patients were followed up, the appetite and physical strength of the patients improved, and ascites decreased or disappeared [Figure 1A (a,b)]. After 4 weeks, the serum biochemical indexes improved gradually and returned to normal. After 12 weeks, the Child–Pugh grade reached grade A. The HIV, HBV, and HCV viral loads were not detected. In the routine therapy group consisting of 7 patients, 6 with nonsurgical treatment died in 2 years due to gastrointestinal bleeding and liver failure, and 1 died of liver failure in the third year.

As shown in Figure 1B, the survival rate was 85.7% in the combination therapy group after 2 years of follow-up, which was significantly higher than 14.3% in the conventional therapy group (*P* = 0.003).

### Effect of combination BMC transplantation with splenectomy on the liver synthesis and secretion

**Figure 2:**
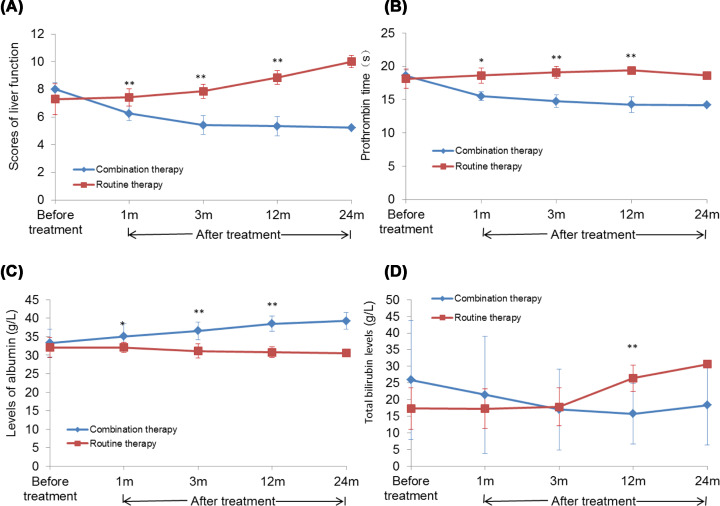
Effect of BMC transplantation on liver synthesis and secretion (**A**) The liver function score before and after the treatment in different groups at different time points. (**B**) The prothrombin time before and after the treatment in different groups at different time points. (**C**) The albumin levels before and after the treatment in different groups at different time points. (**D**) The total bilirubin levels before and after the treatment in different groups at different time points. Compared with the routine therapy group, *P<0.05 and **P<0.01.

As shown in Table 2 and Figure 2A, no significant difference was noted in the liver function scores between the two groups before treatment (*t* = 1.577, *P* = 0.133). However, after treatment, the liver function score in the routine therapy group increased at significantly 1 (*t* = 0.522, *P* = 0.611), 3 (*t* = 1.789, *P* = 0.099), and 12 (*t* = 4.919, *P* = 0.000) months and that in the combination therapy group significantly decreased at 1 (*t* = 4.707, *P* = 0.000), 3 (*t* = 7.216, *P* = 0.000), and 12 (*t* = 7.505, *P* = 0.000) months as compared with that before the treatment. In addition, the liver function score in the combination therapy group was significantly lower than that in the routine therapy group at 1 (*t* = 4.276, *P* = 0.000), 3 (*t* = 9.153, *P* = 0.000), and 12 (*t* = 13.536, *P* = 0.000) months.

**Table 2:** Liver function score before and after the treatment in different groups at various time points

Patient number	Combination therapy						Routine therapy					
	Age (years)	Before treatment	After treatment (months)				Age (years)	Before treatment	After treatment (months)			
			1	3	12	24			1	3	12	24
1	56	8	6	6	5	5	54	8	8	8	9	Death
2	36	8	6	6	5	5	38	8	8	8	9	Death
3	32	9	6	6	6	6	49	7	7	7	8	Death
4	27	7	6	5	5	5	36	7	7	8	9	Death
5	41	10	7	6	6	6	54	7	8	9	10	10
6	48	8	6	5	5	5	48	7	7	8	8	Death
7	35	8	6	5	5	5	39	7	7	7	9	Death
8	39	7	6	5	5	5						
9	26	7	6	5	5	5						
10	54	10	8	6	6	6						
11	54	7	6	5	6	6						
12	36	7	6	5	5	5						

In the liver synthesis function, the prothrombin time (*t* = 0.696, *P* = 0.495) and albumin (*t* = 0.743, *P* = 0.467) level before treatment did not differ significantly between the two groups. After treatment, the prothrombin time decreased significantly, while the albumin level increased significantly at 1 (*t* = 6.279, *P* = 0.000; *t* = 1.328, *P* = 0.196), 3 (*t* = 8.608, *P* = 0.000; *t* = 2.740, *P* = 0.011), and12 (*t* = 11.144, *P* = 0.000; *t* = 4.523, *P* = 0.000) months, respectively as compared with those before the treatment. Moreover, the prothrombin time also decreased significantly and the albumin level increased significantly at 1 (*t* = 6.692, *P* = 0.000; *t* = 2.853, *P* = 0.011), 3 (*t* = 10.379, *P* = 0.000; *t* = 5.185, *P* = 0.000), and 12 (*t* = 15.583, *P* = 0.000; *t* = 8.884, *P* = 0.000) months, respectively that in the routine therapy group (Tables 3 and 4 and Figure 2B,C).

**Table 3:** Prothrombin time (s) before and after the treatment in different groups at various time points

Patient number	Combination therapy						Routine therapy					
	Age (years)	Before treatment	After treatment (months)				Age (years)	Before treatment	After treatment (months)			
			1	3	12	24			1	3	12	24
1	56	17	15.2	14	14.2	13.6	54	17	18.1	17.4	20.2	Death
2	36	19.1	14.8	13.6	14	14.1	38	18.4	18.9	19.2	18.4	Death
3	32	17.9	16	14.4	13.8	14.2	49	18.1	18.4	18.1	19.6	Death
4	27	18.3	14.1	14.6	14.2	14.1	36	17.8	17.9	19.6	19.8	Death
5	41	22.1	17.9	16.2	14.4	14.6	54	19.6	19.8	20.1	17.8	18.6
6	48	19.1	16	15.2	14.9	14.7	48	17.4	18.9	19.4	21.2	Death
7	35	19.1	14.6	14.3	14.1	14.1	39	18.7	18.4	19.6	18.6	Death
8	39	17.1	15.8	14.9	14.2	14.3						
9	26	17.6	14.6	14	14	13.8						
10	54	19.6	17.2	16.4	14.1	14.1						
11	54	17.4	15.3	15.1	14.8	14.8						
12	36	18,3	14.6	14.1	14.2	14.2						

**Table 4:** Albumin levels (g/l) before and after the treatment in different groups at various time points

Patient number	Combination therapy						Routine therapy					
	Age (years)	Before treatment	After treatment (months)				Age (years)	Before treatment	After treatment (months)			
			1	3	12	24			1	3	12	24
1	56	30.8	34.1	34.6	38.6	40.1	54	30.2	30.8	30.6	28.8	Death
2	36	33.5	35.6	36.8	38.8	38.9	38	32	31.6	30.2	31.6	Death
3	32	27.4	38.2	37.9	38.1	38.8	49	34.6	32.8	32.1	29.8	Death
4	27	36.3	36.8	38.6	39.6	41.1	36	31.8	32.6	31.2	31.7	Death
5	41	31.3	28.6	34.2	38.2	38.1	54	29.6	30.9	29.1	31.2	30.6
6	48	36.2	34.8	34.6	34.8	34.1	48	36.8	34.6	34.9	32.8	Death
7	35	37	39.4	36.3	39.1	41.2	39	29.6	31.2	29.8	29.6	Death
8	39	34.1	38.4	39.2	39.4	40.2						
9	26	36.4	33.9	37.3	40	41						
10	54	28.5	30.9	31.6	34.2	34.2						
11	54	38.8	39.1	39.8	41.2	41.2						
12	36	29.1	31.5	37.9	39.8	39.8						

As shown in Table 5 and Figure 2D, the total bilirubin levels in the liver secretion function were not significantly different between the two groups before the treatment (*t* = 1.612, *P* = 0.124). However, after the treatment, the levels decreased significantly in the combination therapy group at 1 (*t* = 0.013, *P* = 0.990), 3 (*t* = 0.147, *P* = 0.885), and 12 (*t* = 3.263, *P* = 0.007) months as compared with those before the treatment. The levels of total bilirubin in the combination therapy group were significantly lower as compared with those in the routine therapy group at 12 months (*t* = 3.758, *P* = 0.001).

**Table 5:** Total bilirubin levels (g/l) before and after the treatment in different groups at various time points

Patient number	Combination therapy						Routine therapy					
	Age (years)	Before treatment	After treatment (months)				Age (years)	Before treatment	After treatment (months)			
			1	3	12	24			1	3	12	24
1	56	12.9	13.6	16.4	16.1	15,2	54	12.9	13.1	12.9	26.8	Death
2	36	48.2	38.6	31.6	28.4	26.4	38	20.8	22.1	23.3	31.6	Death
3	32	56.9	38.9	32.4	26.8	31.2	49	19.2	18.4	19.8	29.4	Death
4	27	16.7	18.9	18.1	17.9	16.6	36	13.5	13.8	14.2	21.8	Death
5	41	57.5	65.5	42.6	31.5	37.5	54	14.7	13.8	14.6	22.1	30.6
6	48	10.5	10.0	9.6	7.8	6.1	48	11.1	11.9	12.6	23.8	Death
7	35	21.8	19.2	8.5	17.0	11.2	39	29.1	27.9	27.2	29.4	Death
8	39	19.2	16.4	14.2	14.4	10.6						
9	26	13.5	5.8	6.1	6.1	6.8						
10	54	14.7	11.4	11.2	10.6	10.8						
11	54	11.1	10.8	4.9	5.8	5.9						
12	36	28	7.6	8.2	6.8	6.8						

### Effect of combination BMC transplantation with splenectomy on differential blood count

**Figure 3:**
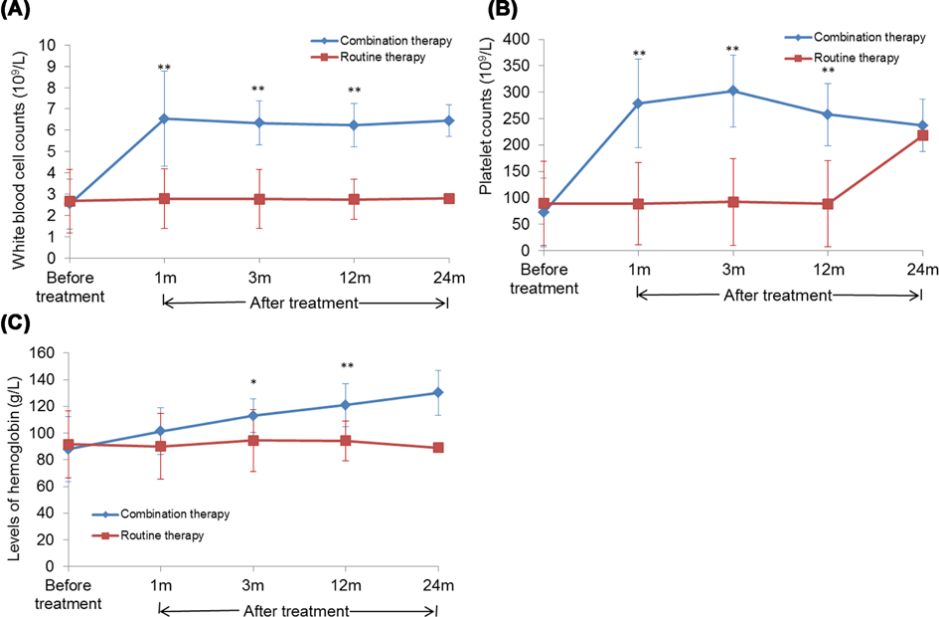
Effect of BMC transplantation on differential blood count (**A**) The WBC counts before and after the treatment in different groups at different time points. (**B**) The platelet counts before and after the treatment in different groups at different time points. (**C**) The hemoglobin levels before and after the treatment in different groups at different time points. Compared with the routine therapy group, *P<0.05 and **P<0.01.

As shown in Tables 6–8 and Figure 3A–C, no significant difference was detected in the WBC counts (*t* = 0.233, *P* = 0.819), hemoglobin levels (*t* = 0.321, *P* = 0.752), and platelet counts (*t* = 0.520, *P* = 0.609) between the two groups before the treatment. After 1, 3, and 12 months of treatment, the WBC counts (*t*_1m_ = 5.946, *P*_1m_ = 0.000; *t*_3m_ = 9.093, *P*_3m_ = 0.000; *t*_12m_ = 8.921, *P*_12m_ = 0.000, respectively), hemoglobin levels (*t*_1m_ = 1.684, *P*_1m_ = 0.104; *t*_3m_ = 3.414, *P*_3m_ = 0.002; *t*_12m_ = 4.235, *P*_12m_ = 0.000, respectively), and platelet counts (*t*_1m_ = 7.247, *P*_1m_ = 0.000; *t*_3m_ = 9.155, *P*_3m_ = 0.000; *t*_12m_ = 7.912, *P*_12m_ = 0.000, respectively) were significantly higher in the combination therapy group than those before the treatment Also, the WBC counts (*t*_1m_ = 4.043, *P*_1m_ = 0.001; *t*_3m_ = 6.712, *P*_3m_ = 0.000; *t*_12m_ = 7.570, *P*_12m_ = 0.000, respectively), hemoglobin levels (*t*_1m_ = 1.222, *P*_1m_ = 0.237; *t*_3m_ = 2.412, *P*_3m_ = 0.026; *t*_12m_ = 3.687, *P*_12m_ = 0.002, respectively), and platelet counts (*t*_1m_ = 4.979, *P*_1m_ = 0.000; *t*_3m_ = 6.255, *P*_3m_ = 0.000; *t*_12m_ = 5.460, *P*_12m_ = 0.000, respectively) were increased as compared with those in the routine therapy treatment group.

**Table 6:** White blood cell count (×10^9^/l) before and after the treatment in different groups at various time points

Number	Combination therapy						Routine therapy					
	Age (years)	Before treatment	After treatment (months)				Age (years)	Before treatment	After treatment (months)			
			1	3	12	24			1	3	12	24
1	56	2.1	6.6	5.4	5.1	6.2	54	2.1	1.9	2.0	2.1	Death
2	36	1.9	7.6	6.6	6.4	5.4	38	3.4	3.4	3.2	3.2	Death
3	32	2.1	3.9	5.4	5.8	5.6	49	5.6	5.4	5.4	4.6	Death
4	27	2.0	8.9	7.1	6.9	7.7	36	1.3	1.1	1.3	2.1	Death
5	41	2.5	11.4	8.6	7.6	6.9	54	2.8	2.9	2.9	2.6	2.8
6	48	2.6	6.8	6.5	6.3	6.8	48	2.2	2.9	3.0	2.8	Death
7	35	3.4	5.1	7.4	7.9	6.8	39	1.3	1.9	1.6	1.9	Death
8	39	5.6	5.4	6.4	7.2	6.8						
9	26	1.1	8.5	6.6	6.3	5.8						
10	54	2.8	4.0	4.9	5.0	4.9						
11	54	3.0	5.2	5.5	5.1	5.3						
12	36	1.3	5.1	5.7	5.2	5.2						

**Table 7:** Platelet count (×10^9^/l) before and after the treatment in different groups at various time points

Number	Combination therapy						Routine therapy					
	Age (years)	Before treatment	After treatment (months)				Age (years)	Before treatment	After treatment (months)			
			1	3	12	24			1	3	12	24
1	56	36	306	286	255	268	54	35	36	38	32	Death
2	36	27	296	326	284	246	38	16	17	18	16	Death
3	32	101	412	346	246	289	49	133	128	131	98	Death
4	27	56	419	427	310	176	36	36	38	34	36	Death
5	41	28	177	286	288	263	54	235	232	241	221	218
6	48	32	177	196	208	212	48	128	131	142	182	Death
7	35	16	153	187	95	142	39	43	41	42	38	Death
8	39	133	312	342	286	268						
9	26	36	258	308	286	268						
10	54	235	248	269	248	242						
11	54	128	296	289	292	266						
12	36	43	290	370	298	274						

**Table 8:** Hemoglobin levels (g/l) before and after the treatment in different groups at various time points

Number	Combination therapy						Routine therapy					
	Age (years)	Before treatment	After treatment (months)				Age (years)	Before treatment	After treatment (months)			
			1	3	12	24			1	3	12	24
1	56	107	109	114	126	132	54	107	108	104	98	Death
2	36	89	103	129	132	112	38	125	119	128	118	Death
3	32	63	89	121	131	134	49	60	62	69	86	Death
4	27	55	94	118	121	152	36	115	114	118	106	Death
5	41	87	109	112	118	143	54	84	78	89	92	89
6	48	120	129	119	121	105	48	86	91	84	88	Death
7	35	125	130	119	157	152	39	64	59	69	72	Death
8	39	60	88	96	112	121						
9	26	115	103	114	116	121						
10	54	84	68	92	96	102						
11	54	86	91	94	98	106						
12	36	64	104	128	124	128						

### Effect of combination BMC transplantation with splenectomy on the serum levels of CD4+ and CD8+ *in vivo*

**Figure 4:**
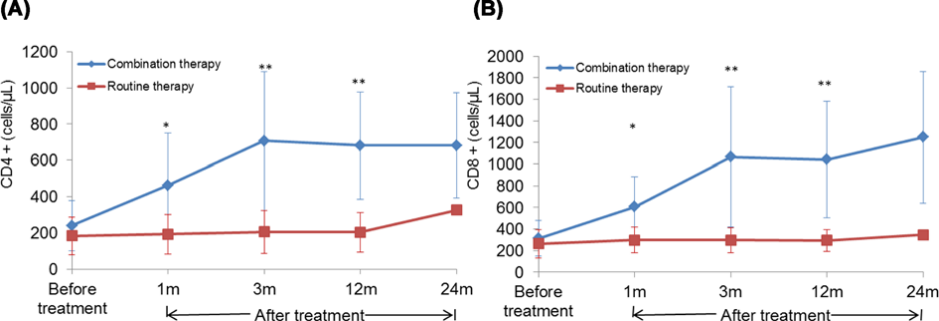
Effect of BMC transplantation on the serum levels of CD4+ and CD8+ *in vivo* (**A**) The levels of CD4+ before and after the treatment in different groups at different time points. (**B**) The levels of CD8+ before and after the treatment in different groups at different time points. Compared with the routine therapy group, *P<0.05 and **P<0.01.

No significant difference was detected in the levels of CD4+ and CD8+ between the two groups before the treatment. After 1, 2, and 12 months of treatment, the levels of CD4+ (*t*_1m_ = 2.630, *P*_1m_ = 0.014; *t*_3m_ = 4.285, *P*_3m_ = 0.000; *t*_12m_ = 5.061, *P*_12m_ = 0.000, respectively) and CD8+ (*t*_1m_ = 3.356, *P*_1m_ = 0.002; *t*_3m_ = 4.212, *P*_3m_ = 0.000; *t*_12m_ = 4.814, *P*_12m_ = 0.000, respectively) were significantly higher those before the treatment in the combination therapy group. Also, the levels of CD4+ (*t*_1m_ = 2.385, *P*_1m_ = 0.028; *t*_3m_ = 3.335, *P*_3m_ = 0.004; *t*_12m_ = 4.090, *P*_12m_ = 0.001, respectively) and CD8+ (*t*_1m_ = 2.778, *P*_1m_ = 0.012; *t*_3m_ = 3.077, *P*_3m_ = 0.006; *t*_12m_ = 3.590, *P*_12m_ = 0.002, respectively) in the combination therapy group were significantly higher than those in the routine therapy group (Tables 9 and 10, Figure 4A,B).

**Table 9:** Levels of CD4+ (cells/µl) before and after the treatment in different groups at various time points

Patient number	Combination therapy						Routine therapy					
	Age (years)	Before treatment	After treatment (months)				Age (years)	Before treatment	After treatment (months)			
			1	3	12	24			1	3	12	24
1	56	61	81	269	341	512	54	58	56	61	68	Death
2	36	80	120	366	519	541	38	86	94	80	90	Death
3	32	130	244	411	612	636	49	210	240	238	238	Death
4	27	265	289	999	1112	1300	36	118	128	146	146	Death
5	41	141	235	326	476	467	54	349	368	398	366	326
6	48	167	481	496	466	492	48	206	198	228	228	Death
7	35	303	864	1087	772	802	39	266	265	289	289	Death
8	39	491	768	1127	982	964						
9	26	157	524	642	395	432						
10	54	387	357	574	546	552						
11	54	384	709	698	686	664						
12	36	313	895	1487	1268	1216						

**Table 10:** Levels of CD8+ (cells/µl) before and after the treatment in different groups at different time points

Patient number	Combination therapy						Routine therapy					
	Age (years)	Before treatment	After treatment (months)				Age (years)	Before treatment	After treatment (months)			
			1	3	12	24			1	3	12	24
1	56	228	318	368	512	825	54	204	218	236	242	Death
2	36	529	621	686	648	796	38	508	514	536	512	Death
3	32	256	362	418	478	639	49	234	249	268	238	Death
4	27	324	649	1886	1892	2501	36	329	386	332	286	Death
5	41	312	462	542	968	999	54	308	332	318	326	348
6	48	178	962	986	946	874	48	168	189	196	228	Death
7	35	174	269	1486	1828	1648	39	108	198	210	226	Death
8	39	680	826	2327	1876	1768						
9	26	183	1179	1783	1215	1189						
10	54	476	362	727	646	668						
11	54	319	570	612	616	646						
12	36	119	696	993	876	886						

## Discussion

HBV and HCV infections may lead to DLC, which is a critical condition with high morbidity and mortality and without adequate treatment. However, for HIV-infected patients, the condition of the liver might aid the immune reconstitution with ART that could be otherwise damaged. Therefore, no curative treatment for HIV-infected patients with DLC leads to the death of patients from liver failure or opportunistic infection [12]. Hitherto, liver transplantation is the most effective treatment for DLC, which also has several limitations, including the shortage of organ donors and high medical expenses. Therefore, stem cell therapy is an alternative option for liver transplantation [12]. Recent evidence showed that stem cell therapy attenuated the clinical conditions of patients with cirrhosis, and improved the liver functions. Stem cells possess the ability of symmetrical self-renewal and pluripotency. Somatic stem cells harbored transgerm layer differentiation potential for migration to the damaged areas; these cells later developed into tissue-committed stem cells [13–15]. Numerous cytokines and growth factors are secreted by BMCs, which promote their migration into injured tissue and facilitate repair.

Several studies demonstrated that a shortened telomere in the liver cells results in the failure of cell replication during the progress of liver cirrhosis. In mammals, the lost liver cells can be replaced by the remaining hepatocytes. However, sustained injury elicits hepatocyte senescence, which in turn, activates liver progenitor cells, known as oval cells. These oval cells have been reported to up-regulate BM-hematopoietic stem cells (BM-HSCs). Both hepatocytes and cholangiocytes can be derived from BM-HSCs. Cell fusion is a critical mechanism during this process. Additionally, BM-HSCs may also promote the proliferation of endogenous hepatocytes to repair liver injury [16,17]. Therefore, stem cell transplantation has been demonstrated to be effective and safe in the treatment of several liver complications, such as post-hepatic cirrhosis, autoimmune liver disease, and alcoholic liver cirrhosis. The function of the liver can be completely restored after stem cell transplantation for patients with alcoholic liver cirrhosis. However, virus-infected patients may need continuous transplantation to replace the damaged stem cells.

Recently, for autologous BMC transplantation, 100–200 ml of autologous bone marrow was needed to isolate MSCs by gradient centrifugation. Then, the isolated MSCs were injected into the liver via a hepatic artery [18] that supplies <33% of the liver blood within a short circulating time. Moreover, the hepatic portal vein is optimal for stem cell transplantation because of its long circulating time and an extended occupation in the liver. Additionally, high levels of hepatotropic cytokines and nutrients also benefit the maintenance of stem cells. Only a few side effects, such as embolism, are observed owing to the small diameter of BMCs. The present study demonstrated that the survival time in the combination therapy group was significantly longer than that in the routine therapy treatment group. Whether stem cells are effective in treating DLC is controversial. A meta-analysis showed that umbilical cord hepatocytes improve the liver function, relieve clinical symptoms, and improve the quality of life in patients with DLC as compared with the traditional supportive care [19]. Another meta-analysis found that tissue-derived stem cell treatment improves the liver function without complications; however, no difference is detected in the liver function and survival as compared with routine therapy [20]. A randomized controlled study in 2018 found that granulocyte colony-stimulating factor with or without hemopoietic stem-cell infusion did not improve liver dysfunction or fibrosis and might be associated with increased frequency of adverse events as compared with routine treatment [21]. The current study found that splenectomy combined with BMC transplantation through the portal vein not only improves the liver function but also improves the 2-year survival rate. Whether or not splenectomy is valuable for the body’s immune reconstruction needs further investigation. Therefore, combination BMC transplantation with splenectomy is an effective technique for the infusion and growth of stem cells in the liver.

BM contains MSCs, HSCs, vascular progenitor cells, many precursor cells, and cytokines. Previous studies demonstrated that pulmonary embolism might occur in patients with multiple fractures due to the infiltration of BM into the venous system [22,23]. However, this embolism might be caused by the massive release of tissue factors and adipocytes from the sites of fracture. The capillary network between the hepatic portal vein and hepatic vein can eliminate components such as adipocytes, avoiding the occurrence of embolism during BMC transplantation. The present *in vivo* study did not detect any fat embolism in the hepatic portal venous system and the lung after the infusion of autologous BMCs at different time points. Moreover, the results demonstrated that patients who underwent autologous BMC transplantation combined with splenectomy exhibited normalized WBCs and platelet counts, elimination of ascites, and restored liver function. All patients were classified as Child–Pugh class A, suggesting that BMC infusion improve liver regeneration.

Three months after splenectomy and autologous BMC infusion, the number of CD4+ T lymphocytes was >300 cells/µl in the patients with no side effects. The liver functions were restored in 12 with no abnormality in the differential blood count. The CD4+ T lymphocytes of these patients were stabilized at >500 cells/µl. Splenectomy is known to avoid spleen-induced damage of various blood cells, leading to rapid repair of WBCs, red blood cells, and platelets. BMCs drive the localization, differentiation, and proliferation of liver-specific cells to renew the liver function [24]. However, CD4+ T lymphocytes are commonly developed in the thymus from precursor T lineage cells migrated from the BM. Then, the mature CD4+ T cells are released into peripheral blood [25]. The application of ART in HIV-infected patients helps to rebuild the immune system with an increased number of CD4+ T cells. In elderly patients, the number of CD4+ T cells increases slowly due to the involution of thymus. The present study demonstrated that the number of CD4+ T cells increased rapidly after autologous BMC transplantation in the 12 HIV-infected patients with liver cirrhosis, which was faster in the younger patients as compared with the elderly. The precursor T lineage cells derived from the infused BMC were implicated as a critical tool for treating HIV-infected patients. Therefore, splenectomy and autologous BMC infusion promote cellular immunity in patients with HIV infection and liver cirrhosis.

## Conclusions

Splenectomy, combined with autologous BMC infusion through the portal vein, is beneficial to HIV-infected patients with DLC. It promotes the recovery of liver function and cellular immune function but may increase the risk of severe complications after the surgery.

## Data Availability

All data generated and/or analyzed in this study are included in this manuscript.

## Abbreviations

ART: antiretroviral therapy
BMC: bone marrow cell
DLC: decompensated liver cirrhosis
HBV: hepatitis B virus
HCV: hepatitis C virus
HIV: human immunodeficiency virus
MSC: mesenchymal stem cell
